# Longitudinal structural and perfusion MRI enhanced by machine learning outperforms standalone modalities and radiological expertise in high-grade glioma surveillance

**DOI:** 10.1007/s00234-021-02719-6

**Published:** 2021-05-28

**Authors:** Loizos Siakallis, Carole H. Sudre, Paul Mulholland, Naomi Fersht, Jeremy Rees, Laurens Topff, Steffi Thust, Rolf Jager, M. Jorge Cardoso, Jasmina Panovska-Griffiths, Sotirios Bisdas

**Affiliations:** 1 Lysholm Department of Neuroradiology, National Hospital for Neurology and Neurosurgery, Queen Square, London, WC1N 3BG UK; 2 Translational Imaging Group, Centre for Medical Image Computing, University College London , London, UK; 3 Department of Medical Physics, University College London, London, UK; 4 Department of Oncology, University College London Hospitals NHS Foundation Trust, London, UK; 5 Department of Brain Repair and Rehabilitation, UCL Institute of Neurology, London, UK; 6 Department of Neurooncology, National Hospital for Neurology and Neurosurgery, London, UK; 7 Department of Radiology, The Netherlands Cancer Institute, Amsterdam, Netherlands; 8 Institute for Global Health, University College London, London, UK; 9 The Queen’s College, University of Oxford, Oxford, UK

**Keywords:** Machine learning, Glioblastoma (GB), Glioma, Radiomics, Perfusion

## Abstract

**Purpose:**

Surveillance of patients with high-grade glioma (HGG) and identification of disease progression remain a major challenge in neurooncology. This study aimed to develop a support vector machine (SVM) classifier, employing combined longitudinal structural and perfusion MRI studies, to classify between stable disease, pseudoprogression and progressive disease (3-class problem).

**Methods:**

Study participants were separated into two groups: group I (total cohort: 64 patients) with a single DSC time point and group II (19 patients) with longitudinal DSC time points (2-3). We retrospectively analysed 269 structural MRI and 92 dynamic susceptibility contrast perfusion (DSC) MRI scans. The SVM classifier was trained using all available MRI studies for each group. Classification accuracy was assessed for different feature dataset and time point combinations and compared to radiologists’ classifications.

**Results:**

SVM classification based on combined perfusion and structural features outperformed radiologists’ classification across all groups. For the identification of progressive disease, use of combined features and longitudinal DSC time points improved classification performance (lowest error rate 1.6%). Optimal performance was observed in group II (multiple time points) with SVM sensitivity/specificity/accuracy of 100/91.67/94.7% (first time point analysis) and 85.71/100/94.7% (longitudinal analysis), compared to 60/78/68% and 70/90/84.2% for the respective radiologist classifications. In group I (single time point), the SVM classifier also outperformed radiologists’ classifications with sensitivity/specificity/accuracy of 86.49/75.00/81.53% (SVM) compared to 75.7/68.9/73.84% (radiologists).

**Conclusion:**

Our results indicate that utilisation of a machine learning (SVM) classifier based on analysis of longitudinal perfusion time points and combined structural and perfusion features significantly enhances classification outcome (*p* value= 0.0001).

**Supplementary Information:**

The online version contains supplementary material available at 10.1007/s00234-021-02719-6.

## Introduction

High-grade gliomas (HGGs) are the most common type of malignant primary brain tumours, representing 80% of newly diagnosed cases, the majority of which (>50%) correspond to glioblastoma (GB) [1, 2]. Current reference standard treatment includes maximal safe resection, radiation therapy and concurrent temozolomide (TMZ) [2, 3]. Poor prognosis and heterogeneous response to treatment warrant imaging surveillance for these patients [4].

Differentiation of progressive disease (PD) from pseudoprogression (PsP) remains critical for patient management [5]. Structural MRI, even under evolving diagnostic criteria, has been inefficient to reliably differentiate PD from PsP [2, 5–7]. Perfusion MRI has been previously shown to improve this classification [8–10]. Studies aiming to differentiate PD from PSP using dynamic susceptibility contrast (DSC) MR perfusion with a single perfusion time point produced initially promising, albeit conflicting results [8, 10]. A recent meta-analysis on the differentiation between PD and PsP by DSC MR perfusion indicates a pooled sensitivity and specificity of 90% and 88% respectively [11].

The combination of perfusion and structural metrics with the use of multiparametric MRI has been successfully applied to differentiate between PD and PsP in treated GBs [12], to predict the location of recurrence postoperatively [13] and to enhance prediction of overall survival [14]. The addition of perfusion time points combined with multiparametric histogram analysis has been shown to enhance prediction of tumour progression and survival [9]. Similarly, longitudinal DSC perfusion assessment for glioma surveillance has been previously shown to identify malignant transformation of low-grade gliomas [15] including oligodendrogliomas [16, 17]. Studies have applied longitudinal MR perfusion to differentiate between PD and PsP, demonstrating that DSC perfusion parameters such as relative cerebral blood volume (rCBV) are potentially superior to conventional MRI parameters such as trends in enhancing tumour volume [18, 19].

The analysis of multiparametric MRI datasets has been further enhanced by the use of machine learning. Such techniques have been successfully employed for the differentiation of PsP from tumour recurrence in patients with resected GB [20, 21], glioma classification by grade and mutation status [22], prediction of overall survival, molecular subtyping of GB [23, 24] and differentiation of tumour from non-tumour components in HGG [25]. Support vector machine (SVM) classifiers have been successfully applied on multiparametric MRI datasets for the classification between PD and PsP based on perfusion MRI [26]. More recent studies demonstrate improved classification accuracy by including perfusion features in multiparametric MRI datasets for radiomic model analysis [27, 28].

Our aim was to comparatively assess the performance of an SVM classifier trained to differentiate PD from PsP for different radiomic feature combinations: (I) combined perfusion and structural radiomic features compared to standalone features and (II) longitudinal compared to single time point radiomic features. Furthermore, we aimed to compare the performance of the SVM classifier against radiologists’ interpretation.

To the best of our knowledge, this is the first study to examine the potential advantage of applying combined structural and perfusion MRI datasets both on single and longitudinal time points, for SVM enhanced classification between PD and PsP in patients with HGG.

## Materials and methods

### Study participants

We retrospectively analysed imaging studies of patients with HGG, investigated in our department with perfusion MRI between 2012 and 2018. Institutional research ethics review approval was obtained. The requirement for informed consent was waived by the UCL research ethics committee. We included patients with histologically confirmed primary diagnosis of HGG, availability of DSC MR perfusion on at least one time point following any treatment and of structural MRI before and after each perfusion time point. We excluded patients who did not have histological confirmation of HGG, had inadequate imaging studies prior and after DSC MR perfusion, or underwent surgery and/or antiangiogenic agent treatment (e.g. bevacizumab) between perfusion time points. Two groups were created for analysis: “group I” consisting of all patients with a single DSC MR perfusion time point (total cohort 64 patients) and “group II” consisting of patients with multiple (2 or 3) DSC MR perfusion time points (19 patients).

### MRI protocol

Structural MRI studies were acquired on a clinical 3T MRI system (Magnetom Prisma Siemens Healthcare, Erlangen, Germany) including the following sequences: T2 fluid-attenuated inversion recovery (FLAIR) [TR/TE/IR 6500/88/2130 ms, slice/gap 5/6.5 mm, field of view (FOV) 165 × 220 mm^2^]; T2WI [TR/TE 4610/99.5, slice/gap 5/1.5 mm, FOV 210 × 210 mm^2^]; T1WI [TR/TE 415/20 ms, slice/gap 5/1.5 mm, FOV 210 × 210 mm^2^]; DWI [TR/TE 3700/55 ms, slice/gap 4/5 mm, acquisition matrix 192×192, FOV 220×220 mm^2^]; T1WI post-contrast (Dotarem, Guerbet, Villepinte, France) [TR/TE 6.8/450 ms, slice/gap 5/6.5 mm, acquisition matrix 256×256, FOV 217×240 mm^2^].

Perfusion-weighted MRI studies were acquired on the same system using a standard dynamic susceptibility weighted contrast perfusion MR imaging protocol consisting of a gradient echo-EPI sequence, TR/TE 1370/30, slice/gap 4/5.2 mm, field of view (FOV) 220x220, acquisition matrix 128x128, flip angle 65, scan time: 2 min 20 s. EPI data were acquired following injection of a 0.1-mmol/kg body weight bolus of gadoterate meglumine (Dotarem, Guerbet, Villepinte, France) followed by a 20-ml bolus of saline, both at a constant rate of 5 ml/s. Pre-load with half-dose gadolinium was applied. All external MRI scans included in the study were based on similar protocols.

### DSC perfusion analysis

Postprocessing of DSC PWI studies was performed using Olea Sphere 3.0 (Olea Sphere®

3.0, Olea Medical®) following correction for patient motion using the built-in software feature. The arterial input function was selected automatically using a cluster analysis algorithm [29] and manually corrected in cases of discrepancy with the anatomical images. Deconvolution-based perfusion parameters were calculated using Bayesian probabilistic methods, following contrast leakage correction with the built-in software feature [30]. Relative cerebral blood volume (rCBV) and relative cerebral blood flow (rCBF) maps were calculated on DSC MR perfusion studies. Normalised values (z-scores) of both rCBV and rCBF were calculated respective to the ipsilateral basal ganglia. Perfusion normalisation with similar methodology has been previously shown to decrease variability of rCBV measurements. Based on previous studies, the basal ganglia have been used for normalisation to allow automated segmentation and reduce variability related to perfusion variations within the white matter and user-dependent selection of regions of interest [31–33].

### Treatment response assessment

Treatment response assessment included the following categories: progressive disease (PD), pseudoprogression (PsP), stable disease (SD), partial response (PR) and complete response (CR). Lesion classification was based on histology following repeat surgery or biopsy where available (13 of a total cohort of 64 cases: 20%). In the remaining cases, classification was based on prolonged radiological and clinical surveillance. Radiological surveillance was based on the course of enhancing lesions on prolonged longitudinal MRI. Clinical surveillance was based on the final outcome of the local neuro-oncology multidisciplinary team meeting which assessed all available clinical and radiological data at the latest available time point. This classification was considered as expert consensus ground truth. No complete response cases were encountered in our cohort. The few preliminary partial response cases encountered (4), converted to other categories during surveillance. Therefore, training of the SVM classifier was based on three categories: PD, PsP and SD (3-class problem).

### Image co-registration, segmentation

**Fig. 1:**
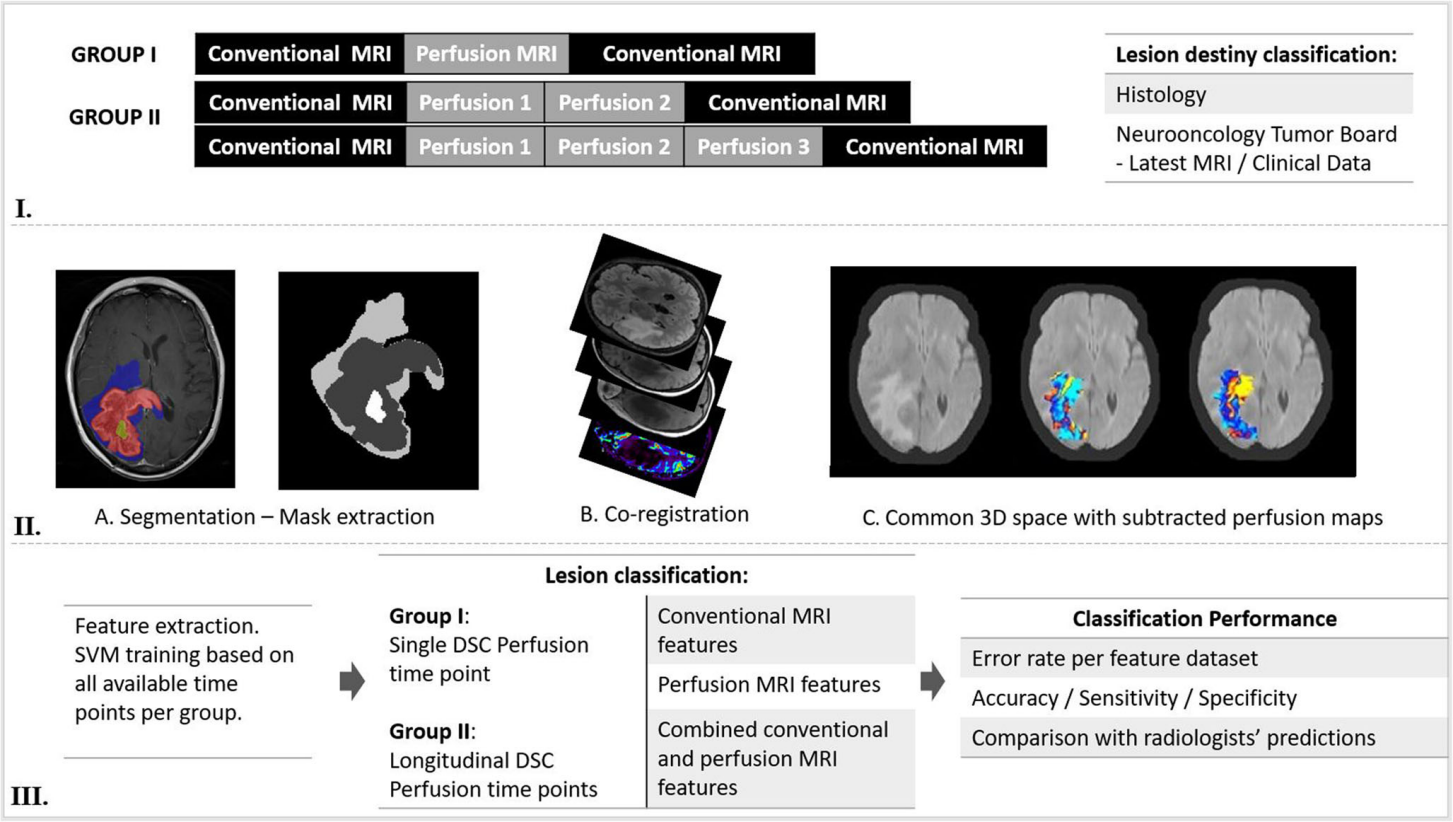
Summary of methodology and radiomics workflow. I. Study participants were separated into two groups: group I with a single DSC MR perfusion time point and group II with multiple (2 or 3) DSC MR perfusion time points. All patients had structural MRI prior and following each perfusion time point. Each imaging time point was classified as progressive disease (PD), pseudoprogression (PsP) or stable disease (SD). Histology was used as ground truth for lesion classification where available. In cases without histological confirmation, lesion classification was based on the final outcome of the local neuro-oncology multidisciplinary team meeting which assessed all available serial radiological surveillance studies as well as clinical data at the latest available time point. This was considered as the expert consensus ground truth. II. Lesion areas were identified and segmented including hyperintensity on FLAIR (blue), contrast enhancement (red) and necrosis (yellow—excluded). Segmentation masks were exported. A common 3D space was created for each patient using axial T1, post contrast T1W and FLAIR images from every time point, following log-transformation, normalisation, bias field correction and intensity matching of the skull-stripped images. The perfusion maps corresponding to the extracted masks were co-registered on the common 3D space. III. Feature extraction and SVM training were based on different combinations of feature datasets (structural, perfusion and combined) and perfusion time points (single, longitudinal). Classification performance was assessed by calculating error rates and accuracy/sensitivity/specificity of classification for each feature dataset. SVM classification results were compared to radiologists’ predictions

Our methodology is outlined in Fig. 1. A common 3D space was created for each patient using axial T1, post contrast T1W and FLAIR images of all time points. This was based on a previously reported method for affine registration following log-transformation, normalisation, bias field correction and intensity matching of the skull-stripped images [34]. Images from all available time points were resampled to a common space and subtraction datasets of normalised maps were created.

Segmentation was conducted by a neuroradiologist with 2 years’ experience (L.S), and results were supervised independently by a neuroradiologist with more than 10 years’ experience in brain tumour imaging (S.B). Areas of enhancing tissue on post-contrast T1W images and non-enhancing T2 hyperintensity were manually segmented for all available time points. Segmentation was based on T2 FLAIR images overlaid on post contrast T1W images, excluding any resection cavities and areas of macroscopic necrosis (www.itksnap.org) [35].

### Feature extraction

The segmentation masks were applied to the common multiparametric space and utilised for feature extraction for all patients. Imaging features were derived from multiple sequences including T1 (pre- and post-contrast), T2, FLAIR, rCBV and rCBF at all available time points. Total extracted features were 130 for the single time point group and 110 for the multiple time point group. For the multiple time point group, subtracted features between different time points were calculated (subtractions included 2-1, 3-2, 3-1). These included longitudinal changes in signal intensity on pre- and post-contrast sequences on structural MRI studies, as well as longitudinal differences in rCBV and rCBF values on consecutive DSC MR perfusion studies. Distribution and textural features over the normalised subtraction images were extracted and used as features for automated classification. A least absolute shrinkage and selection operator (LASSO) framework [36] was applied for feature selection preceding the application of the SVM classifier for automated lesion classification (PD, PsP, SD).

### SVM classifier

A binary SVM with radial basis function (RBF) kernel was constructed using all selected features. The code used to generate results is freely available at https://github.com/csudre/LongPerfusion. The single time point SVM training dataset was based on all available structural and perfusion MRI studies at a single time point. The longitudinal SVM training dataset included all available normalised images form structural and perfusion MRI studies, at every time point (time points 1, 2, and in some cases 3). K-fold cross validation was employed for SVM training and validation, and results were averaged over 250 two-fold cross-validation iterations [37]. Separate classification steps were performed and included “one vs all” dichotomous classifications between: (a) SD vs (PD and PsP) and (b) PD vs (SD and PsP). Final SVM analysis utilised both steps and resulted in a final classification for each case (SD, PD or PsP).

### Diagnostic performance

To assess diagnostic performance, error rates and sensitivity/specificity/accuracy were calculated for SVM classification based on different feature datasets (structural, perfusion, combined), different classification steps and different time points (single and multiple perfusion time points). Comparisons of classification accuracy were performed based on these metrics for both groups.

Consequently, radiological reports were extracted for comparison. These were based on structural and perfusion MRI studies for all patients, reported by a team of three senior neuroradiologists (at least 7 years’ experience in neuro-oncology imaging and core members of the multidisciplinary neuro-oncology board). Radiological reports included assessment of disease status for each time point and formed the basis for “radiologist classification”. Radiologists had access to all available structural and perfusion imaging studies at every time point and were blinded to lesion classification at the time of reporting.

Comparison between radiological and SVM classification performance was based on sensitivity, specificity, and accuracy of classification. Statistical significance was assessed using McNemar’s statistical test based on methodology employed in previous studies [38, 39].

## Results

### Study participants - lesion classification

The final analysed cohort included 64 participants (age 48.5 ± 12.8 [mean, SD], 24 female). All time points were selected following completion of initial chemoradiation therapy. From an initial group of 187 patients with DSC MRI exams, we excluded 123 cases due to histological diagnosis other than high-grade glioma, unavailable or inadequate structural imaging studies prior to and after DSC MR perfusion, or inadequate information on lesion histology and patient treatment. All included patients had histologically proven HGG, at least one DSC MR perfusion time point, as well as structural MRI preceding and following each perfusion time point. The final cohort comprised of 64 patients: 45 cases with a single time point and 19 cases with multiple DSC time points. Study participants were separated into two groups: group I (single DSC time point: 64 patients) and group II (multiple DSC time points: 19 patients). A flowchart outlining patient selection is provided (Supplementary Figure 1).

Patient demographics and clinical characteristics are summarised in Table 1. Lesion histology is available in supplementary material (Supplementary Tables 1 and 2). The single time point group (group I: 64 patients) included 43 patients with GB (WHO grade 4), 14 patients with anaplastic astrocytoma (grade 3) and 7 patients with oligodendroglioma (grade 3). The multiple time point group (group II 19 patients) included 14 patients with GB, 3 patients with anaplastic astrocytoma and 2 patients with anaplastic oligodendroglioma. In total, we included 269 complete structural MRI and 92 DSC MR perfusion studies. The time interval between the initial and final imaging studies during surveillance was (mean, SD [95% CI] days): group I (201, 159 [182–220]), group II (208, 170 [172–243]).

**Table 1:** Patient demographics, tumour histology and lesion classification

Patient population – tumour type	Group II (multiple DSC time points)	Group I (single DSC time point)
Total number of patients	19	64
Sex ratio (M/F)	11/8	40/24
Mean age	45	48.5
Tumour type		
Glioblastoma (WHO grade 4)	13	43
Anaplastic astrocytoma (WHO grade 3)	4	14
Anaplastic oligodendroglioma (WHO grade 3)	2	7
Progressive disease (PD)	8	37
Pseudoprogression (PsP)	5	13
Stable disease (SD)	6	14
Surgical treatment		
Gross total resection, *N* (%)	13 (68%)	38 (60%)
Sub-total resection, *N* (%)	2 (11%)	13 (20%)
No surgery, *N* (%)	4 (21%)	13 (20%)
Follow up interval (days) Mean, SD [95% CI]:	208, 170 [172–243]	201, 159 [182–220]
Follow up interval in days Mean, SD [95% CI] [PsP cases]	272, 218 [149–395]	216, 176 [61–370]

Treatment response assessment and lesion classification are summarised in supplementary material (Supplementary Tables 1 and 2). Lesion classification per group included: group I (single time point): 37 PD, 13 PsP and 14 SD cases and group II (multiple time points): 8 PD, 5 PsP and 6 SD cases.

### SVM feature selection—classification error rates

Classification performance of radiomic features was assessed via an integrative analysis. Features with the highest predicting accuracy were similar in both groups. The best performance was identified for subtracted values of these features in the multiple time point group including differences in the 25th percentile (P25) of rCBF (Diff ZrBF First Quartile), rCBV (Diff ZrBV Correlation), T2 kurtosis (Diff T2 Kurtosis) and subtracted signal intensity on T1 post contrast images (Diff T1Gad sum average).

SVM classification error rates were calculated for both groups following multiple iterations, to allow comparison between different datasets. In an exploratory way, classification performance was also assessed for different combinations of lesion status as follows: (PsP/SD vs PD and SD vs PsP/PD).

Classification results for group I (single time point, 64 patients) are provided in supplementary material (Supplementary Figure 2). In this group, the combination of structural and perfusion features outperformed both standalone perfusion and structural feature datasets for the clinically relevant classification of PD versus PsP/SD (median error rates: combined structural and perfusion features 2%, perfusion features 4%, structural features 15.6%. Mean error rates: 23%, 27% and 28% respectively).

**Fig. 2:**
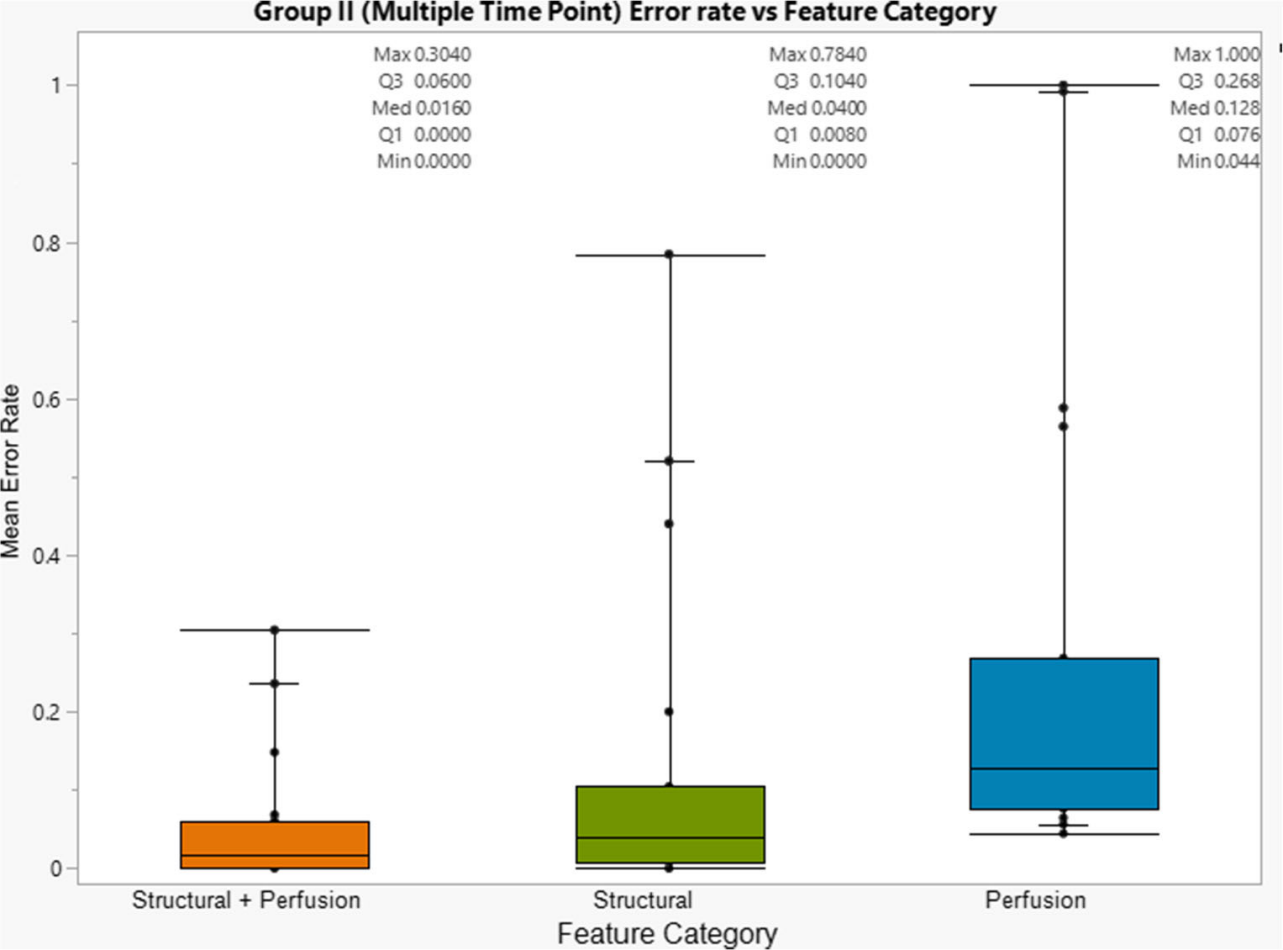
Box plots illustrating the calculated error rate for the clinically relevant SVM classification (SD/PsP vs PD) for group II ( multiple DSC perfusion time points), per feature category. The combination of structural and perfusion features outperformed standalone structural or perfusion feature datasets yielding the lowest classification error rate (median error rate: 1.6%, mean error rate: 5%). Error rate differences were statistically significant (Wilcoxon/Kruskal-Wallis test: *p* value = 0.0001)

Classification in group II (multiple time points, 19 patients) was performed via sampling all combinations of different time points and feature datasets (structural and perfusion) for each time point. When a single perfusion time point was employed (Fig. 2), the combination of perfusion and structural features resulted in lower final classification error rates compared to standalone modalities. Specifically, for the clinically relevant classification of PD vs PSP/SD, the lowest error rate was achieved when the combination of structural and perfusion features was employed (median error rate: 1.6%, mean error rate: 5%).

In group II, classification performance was also assessed for subtracted datasets from different perfusion time point combinations (i.e., first and second time points, first and third time points etc.). Overall, the input of combined perfusion and structural features consistently resulted in lower classification error rates for all time point combinations. Classification based on the first time point resulted in a mean error rate of 10.5%, which was improved to 9.8% when a second time point was introduced (mean interval of 88.2 days). The introduction of an additional, third time point (mean interval of 287.5 days from the initial time point) further reduced the mean error rate to 9.3%. The lowest achievable error rate for stepwise classifications was observed for combined subtracted features between the third and first perfusion time points (median error rate: 0.4%, mean error rate: 0.7%).

### Comparison between radiological reports and SVM-based classification

We calculated sensitivity, specificity and accuracy of lesion classification by radiologists and the SVM classifier. Results are summarised in Table 2. In group II (multiple DSC time points), the SVM outperformed radiologists’ classification. The sensitivity/specificity/accuracy for the SVM classification was 100/91.67/94.7% (analysis based on the first perfusion time point) and 85.71/100/94.7% (longitudinal analysis based on multiple time points) compared to 60/78/68% and 70/90/84.2% for the respective radiologist classifications. In group I (single perfusion time point), the SVM also exceeded radiologist classification performance, albeit by a smaller margin and resulted in sensitivity/specificity/accuracy of 86.49/75.00/81.53% (SVM) compared to 75.7/68.9/73.84% (radiologists). Combined perfusion and structural features consistently outperformed standalone datasets for SVM-based classification for all time point combinations.

**Table 2:** SVM and radiologist classification performance assessment

	SVM				Radiologists	
	Group I (single time point)	Group II (first time point analysis)	Group II (longitudinal analysis)	Group I (single time point)	Group II (first time point analysis)	Group II (longitudinal analysis)
Sensitivity (%)	86.49	100	85.71	75.7	60	70
Specificity (%)	75.00	91.67	100	68.9	78	90
Accuracy (%)	81.53	94.7	94.7	73.84	68	84.2

McNemar’s test, SVM vs radiologist classifications (*p* value): group I: *p* value = 0.041, group II (first time point analysis): *p* value = 0.034, group II (longitudinal analysis): *p* value = 0.025

Statistical assessment using McNemar’s statistical test rejected the null hypothesis of equal performance between the radiologists and the SVM classifier (*p* value < 0.05 for the respective comparisons, provided in Table 2).

## Discussion

This study comparatively assessed the performance of an SVM classifier for the differentiation between PD, SD and PSP during post-treatment surveillance of patients with high-grade glioma (HGG). Our results demonstrate improved SVM classification performance following the application of combined perfusion and structural MRI features and the introduction of longitudinal perfusion time points. Furthermore, our results indicate that the optimal SVM classifier outperforms radiologists’ interpretation in our cohort.

Optimal SVM classification performance was observed when longitudinal perfusion studies were analysed based on combined perfusion and structural features, exceeding radiologists’ classification performance in both patient groups. Applying the classifier across two groups (group I and II with either 1 or 2–3 DSC points respectively), we demonstrate that the sensitivity/specificity/accuracy of the SVM were superior to radiologists’. Specifically, for SVM-based classification, these metrics are 100/91.67/94.7% (first perfusion time point analysis) and 85.71/100/94.7% (longitudinal analysis), compared to 60/78/68% and 70/90/84.2% for the respective radiologists’ classifications. To the best of our knowledge, this is the first study to comparatively assess SVM classification performance based on single and longitudinal perfusion time points using combinations of structural and perfusion feature datasets, and to compare SVM classification performance to that of expert radiologists.

Our findings highlight the potential of multiparametric MRI for determining disease progression and are in accordance with previous studies. Several studies utilised perfusion MRI parameters for discrimination between disease progression and treatment-related effects predominantly evaluating either mean rCBV or maximum rCBV, however producing inconsistent results [8, 40–42]. A recent meta-analysis reports a pooled specificity and sensitivity of 90% and 88% (95% CI 0.85–0.94; 0.83–0.92) for each study’s best-performing DSC MR perfusion parameter [11]. However, in contrast to these studies and similarly to previous studies by Hu et al and Park et al [43], our methodology is not based on predetermined perfusion features or cut-off values during SVM training.

We observed that the combination of perfusion and structural MRI features consistently improved classification performance for both single and longitudinal perfusion time point group analyses. Recent studies examining the identification of pseudoprogression based on a single perfusion time point are consistent with this observation [27, 28, 44].

Extending further than current knowledge, our results indicate that SVM classification based on longitudinal DSC perfusion time points outperforms single time point analysis in predicting lesion destiny, a finding not previously described by similar studies. The longitudinal analysis in our cohort was characterised by improved performance both in terms of classification error rate and sensitivity/specificity/accuracy, although inherent group cofounders may bias direct comparisons.

Specifically, classification performance appeared to be related to the number of the DSC MR perfusion time points. When a single time point was used for SVM training based on combined perfusion and structural features, sensitivity/specificity/accuracy were 86.49/75.00/81.53% respectively (mean error rate 10.5%). The application of a second perfusion time point (mean interval time 88.2 days) resulted in increased classification performance (sensitivity/specificity/accuracy 85.71/100/94.73%, mean error rate 9.8%). The introduction of an additional, third time point (mean interval 287.5 days) resulted in similarly improved classification performance and further reduction in error rate (sensitivity/specificity/accuracy 85.71/100/94.73%, mean error rate 9.3%). The limited number of subjects undergone three longitudinal perfusion scans in our cohort does not allow definite conclusions. However, it is worth noting that the lowest achieved error rate was observed when combined subtracted features of all three time points were applied for SVM classification (median error rate 0.4%, mean error rate 7%).

Another important finding with potential clinical implication is that the SVM outperformed radiologists’ classification performance up to 27% in terms of classification accuracy, more profoundly in the multiple time point group. A similar observation is described in a recent study employing radiomic features derived from structural MRI [45]. However, any generalisation of such comparisons would require confirmation by studies based on larger patient cohorts, prospective design and use of external validation.

The identified difference in performance may be attributed to the ability of the SVM algorithm to detect minute composite differences in time and space using simultaneously perfusion and structural imaging parameters, a time-consuming and challenging task for the reporting radiologist. Indeed, the best-performing features included differences in rCBV, rCBF, T2 kurtosis and subtracted signal intensity on post-contrast T1 sequences on longitudinal MRI studies. Similar features have been identified by previous studies as promising for the differentiation of PsP from PD [44–47]. Employing machine learning, Akbari et al. further demonstrated a correlation between such features, namely enhancement on post-contrast T1 and rCBV, with histologically validated tissue characteristics related to PsP and PD [48]. Differences in contrast enhancement and perfusion metrics are routinely employed by radiologists for lesion characterisation using advanced imaging. However, the incorporation of longitudinal changes of multiparametric MRI features in routine clinical reporting poses a time-consuming and challenging task for human readers.

This study has potential limitations. Importantly, the small patient cohort and retrospective design of the study potentially limit generalisability of our results, which should be validated on larger, well-characterised patient cohorts. To accommodate for the small number of included studies and mitigate potential overfitting, K-fold cross validation was employed to derive training and validation datasets. Histological confirmation of lesion destiny was not available for all patients, however, this is rarely available at multiple imaging time points in HGG patients. Therefore, in such cases, we used expert consensus as ground truth. A similar approach has been almost universally employed in similar studies [19, 25–27, 44, 48]. Potential co-occurrence of viable tumour tissue and radiation necrosis within enhancing lesions poses an additional challenge for the characterisation of treatment response. Prolonged serial imaging was employed to account for this and to allow a more objective final lesion characterisation. A limited number of external scans were included, introducing partial heterogeneity of MRI scan protocols. This potential source of bias is well recognised in clinical practice and was addressed by the construction of a common 3D space. In general, accurate comparison of multiple MRI studies of referred patients which are frequently inconsistent in imaging protocol and quality, dictates the creation of a tool to mitigate any bias and allow assessment of disease evolution despite any technical or quality differences. We believe that our approach is promising to address this need. Overcoming the above limitations is crucial towards clinical application of automated lesion classification based on similar methodologies. Specifically, confirmation of the clinical value of the described approach requires prospective studies incorporating larger patient cohorts, homogeneous scanning parameters, higher percentage of histologically confirmed lesion classifications and external validation of model performance with independent datasets.

In conclusion, our findings indicate that analysis of perfusion and structural MRI data enhanced by machine learning, significantly improves classification between SD, PD and PsP, peaking in performance when multiple perfusion time points are acquired and taken into analysis. This is, to date, the first study designed specifically to allow comparative assessment of classification performance for standalone and combined structural and perfusion MRI features, derived from a single and longitudinal perfusion time points. Automatic classification of lesions by SVM classifiers trained on longitudinal perfusion and structural MRI studies, may also outperform neuroradiological expertise in predicting lesion destiny. Our preliminary findings warrant confirmation by larger, ideally prospective, studies.

## Supplementary information

ESM 1 (JPG 185 kb)

ESM 2 (PNG 600 kb)

High Resolution (TIF 79 kb)

ESM 3 (DOCX 30 kb)

## Abbreviations

PD: Progressive disease
PsP: Pseudoprogression
SD: Stable disease
PR: Partial response
CR: Complete response
rCBV: Relative cerebral blood volume
rCBF: Relative cerebral blood flow
SVM: Support vector machine
HGG: High-grade glioma
GB: Glioblastoma
FLAIR: Fluid-attenuated inversion recovery
