# Supplementary figures and images for: Longitudinal structural and perfusion MRI enhanced by machine learning outperforms standalone modalities and radiological expertise in high-grade glioma surveillance
Source: Neuroradiology. 2021 May 28;63(12):2047–56. doi: 10.1007/s00234-021-02719-6 (PMC8589799; doi:10.1007/s00234-021-02719-6)

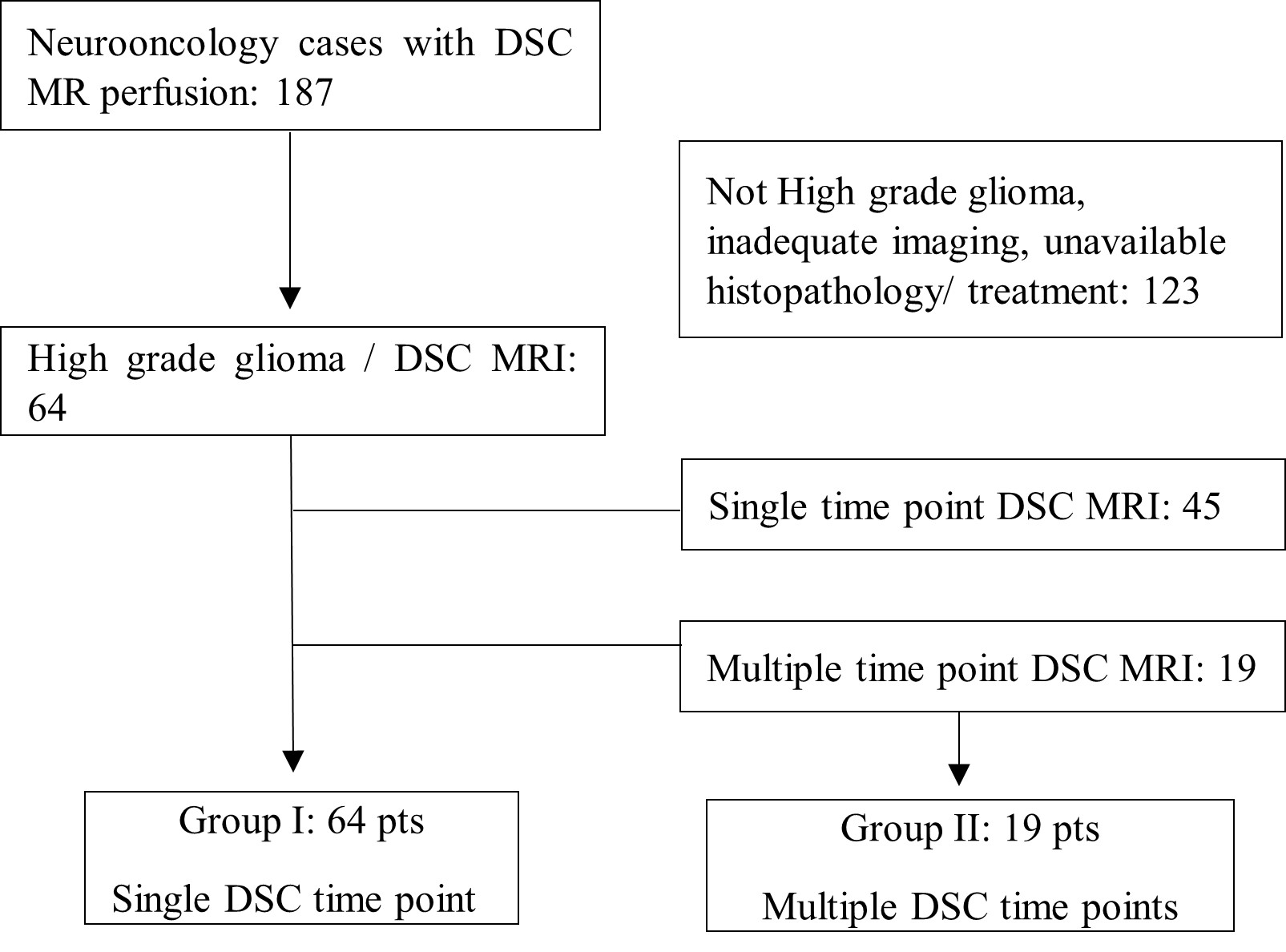

Supplement: Supplementary file 1 — (JPG 185 kb) [file 234_2021_2719_MOESM1_ESM.jpg]

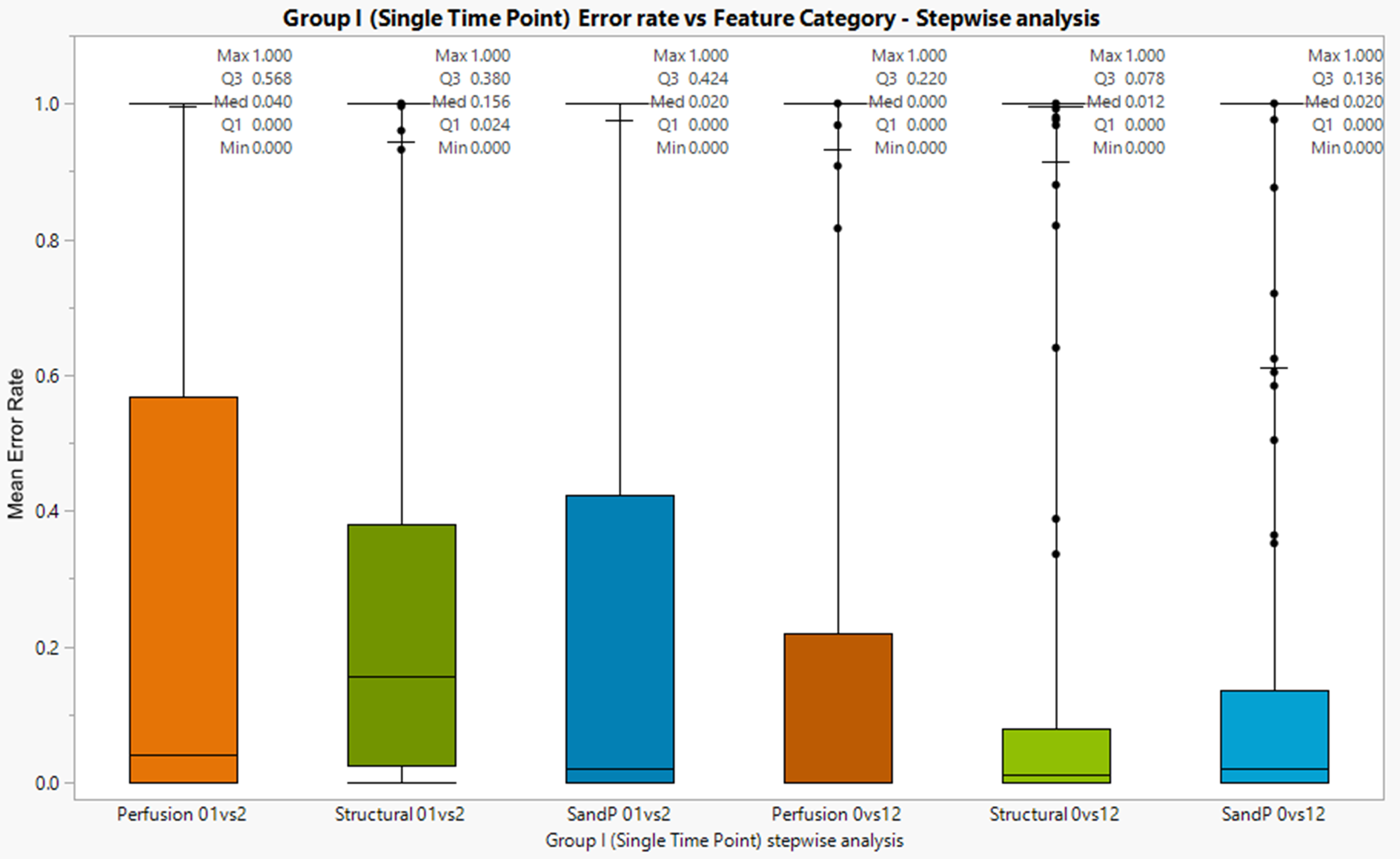

Supplement: Supplementary file 2 — (PNG 600 kb) [file 234_2021_2719_Fig2_ESM.png]

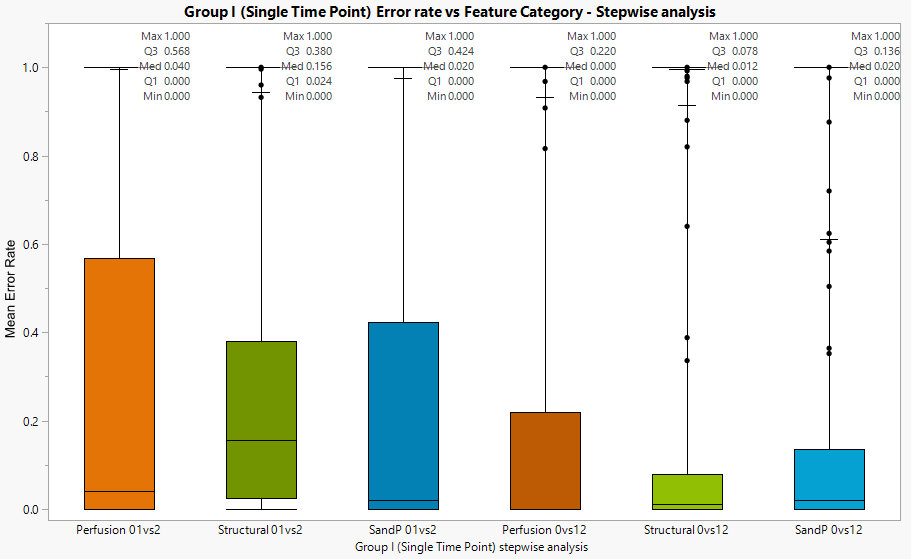

Supplement: Supplementary file 3 — High Resolution (TIF 79 kb) [file 234_2021_2719_MOESM2_ESM.tif]
