# Supplementary material for: Longitudinal structural and perfusion MRI enhanced by machine learning outperforms standalone modalities and radiological expertise in high-grade glioma surveillance
Source: Neuroradiology. 2021 May 28;63(12):2047–56. doi: 10.1007/s00234-021-02719-6 (PMC8589799; doi:10.1007/s00234-021-02719-6)
Supplement: Supplementary file 4 — (DOCX 30 kb) [file 234_2021_2719_MOESM3_ESM.docx]

## Supplementary Material – Figure captions

**Supplementary Fig. 1**. Flowchart outlining patient selection and final included patient cohorts.

**Supplementary Fig. 2**. Box plots illustrating the calculated error rate of SVM classification for Group I (single DSC perfusion time point, 64 patients). Error rates were calculated for each classification step and each feature dataset separately. Lesion categories are coded as: 0: Stable Disease, 1: Pseudoprogression, 2: Progressive Disease. For the clinically relevant classification step (SD/PsP vs PD: 01vs2), the lowest prediction rate was observed when combined structural and perfusion (S and P) feature datasets were utilised (median error rate: 2%, mean error rate: 23%). Standalone structural and perfusion features yielded decreased accuracy with higher classification error rates: structural features (median error rate: 15%, mean error rate: 27%), perfusion features (median error rate: 4%, mean error rate: 28%). For the less clinically relevant classification step (SD vs PSP/PD: 0vs12), lower median error rates were produced for all feature datasets. Error rate differences were statistically significant (Wilcoxon/Kruskal-Wallis test: p value = 0.0002).

## Supplementary Material - Tables

**Supplementary Table 1**. Histological type and lesion classification : Group I.

| Group I: Single DSC MR perfusion time point (all patients) | | | | | |
| --- | --- | --- | --- | --- | --- |
| Patient | Age | Sex | Tumor type | WHO grade | Lesion classification |
|  | 45 | M | Anaplastic Oligodendroglioma | 3 | Stable |
|  | 29 | F | Anaplastic Astrocytoma | 3 | Progression |
|  | 55 | M | Anaplastic Astrocytoma | 3 | Progression |
|  | 41 | F | GB | 4 | Progression |
|  | 56 | F | GB | 4 | Progression |
|  | 77 | F | GB | 4 | Progression |
|  | 40 | M | GB | 4 | Progression |
|  | 36 | F | Anaplastic Oligodendroglioma | 3 | Progression |
|  |  |  | GB | 4 | Stable |
|  | 55 | M | Anaplastic Oligodendroglioma | 3 | Progression |
|  | 42 | M | Anaplastic Astrocytoma | 3 | Pseudoprogression |
|  | 35 | M | Anaplastic Astrocytoma | 3 | Stable |
|  | 39 | M | GB | 4 | Stable |
|  | 50 | M | GB | 4 | Pseudoprogression |
|  | 47 | M | GB | 4 | Pseudoprogression |
|  | 65 | M | GB | 4 | Progression |
|  | 46 | M | GB | 4 | Progression |
|  | 43 | F | Anaplastic Astrocytoma | 3 | Progression |
|  | 58 | M | GB | 4 | Progression |
|  | 55 | F | GB | 4 | Stable |
|  | 57 | F | GB | 4 | Progression |
|  | 40 | M | GB | 4 | Progression |
|  | 33 | M | Anaplastic Astrocytoma | 3 | Progression |
|  | 41 | M | Anaplastic Astrocytoma | 3 | Pseudoprogression |
|  | 45 | M | GB | 4 | Progression |
|  | 68 | F | GB | 4 | Progression |
|  | 60 | F | Anaplastic Astrocytoma | 3 | Progression |
|  | 36 | M | Anaplastic Astrocytoma | 3 | Stable |
|  | 60 | M | GB | 4 | Progression |
|  | 32 | M | Anaplastic Oligodendroglioma | 3 | Stable |
|  | 62 | M | Anaplastic Oligodendroglioma | 3 | Progression |
|  | 71 | F | GB | 4 | Progression |
|  | 43 | M | GB | 4 | Progression |
|  | 52 | M | GB | 4 | Progression |
|  | 44 | M | GB | 4 | Progression |
|  | 45 | F | GB | 4 | Progression |
|  | 55 | F | GB | 4 | Pseudoprogression |
|  | 52 | M | GB | 4 | Stable |
|  | 41 | F | Anaplastic Astrocytoma | 3 | Pseudoprogression |
|  | 64 | M | GB | 4 | Progression |
|  | 58 | F | GB | 4 | Progression |
|  | 52 | M | GB | 4 | Progression |
|  | 67 | F | GB | 4 | Progression |
|  | 47 | M | GB | 4 | Pseudoprogression |
|  | 52 | M | GB | 4 | Pseudoprogression |

**Supplementary Table 2**. Histological type and lesion classification : Group II.

| Group II: Longitudinal DSC MR perfusion time points | | | | | |
| --- | --- | --- | --- | --- | --- |
| Patient | Age | Sex | Tumor type | WHO grade | Lesion classification |
|  | 41 | F | Anaplastic Oligodendroglioma | 3 | Stable |
|  | 39 | F | Anaplastic Oligodendroglioma | 3 | Stable |
|  | 38 | M | GB | 4 | Stable |
|  | 47 | F | GB | 4 | Progression |
|  | 37 | F | GB | 4 | Progression |
|  | 46 | M | GB | 4 | Progression |
|  | 58 | M | GB | 4 | Progression |
|  | 70 | M | GB | 4 | Progression |
|  | 38 | F | Anaplastic Astrocytoma | 3 | Pseudoprogression |
|  | 49 | F | Anaplastic Astrocytoma | 3 | Stable |
|  | 41 | M | Anaplastic Astrocytoma | 3 | Pseudoprogression |
|  | 66 | F | GB | 4 | Progression |
|  | 56 | M | GB | 4 | Pseudoprogression |
|  | 23 | M | GB | 4 | Stable |
|  | 63 | M | GB | 4 | Pseudoprogression |
|  | 56 | M | GB | 4 | Progression |
|  | 54 | M | GB | 4 | Stable |
|  | 35 | M | GB | 4 | Progression |
|  | 41 | M | GB | 4 | Progression |
